# Donor-Specific Cell-Based Assays in Studying Sensitivity to Low-Dose Radiation: A Population-Based Perspective

**DOI:** 10.3389/fpubh.2014.00244

**Published:** 2014-11-18

**Authors:** Dora Il’yasova, Alexander Kinev, C. David Melton, Faith G. Davis

**Affiliations:** ^1^Division of Epidemiology and Biostatistics, School of Public Health, Georgia State University, Atlanta, GA, USA; ^2^Creative Scientist, Inc., Durham, NC, USA; ^3^School of Public Health, University of Alberta, Edmonton, AB, Canada

**Keywords:** low-dose ionizing radiation, individual variability, biomarkers, epidemiology

## Abstract

Currently, a linear no-threshold model is used to estimate health risks associated with exposure to low-dose radiation, a prevalent exposure in the general population, because the direct estimation from epidemiological studies suffers from uncertainty. This model has been criticized based on unique biology of low-dose radiation. Whether the departure from linearity is toward increased or decreased risk is intensely debated. We present an approach based on individual radiosensitivity testing and discuss how individual radiosensitivity can be assessed with the goal to develop a quantifiable measure of cellular response that can be conducted via high-throughput population testing.

## Why Do We Need A New Approach?

High-dose ionizing radiation (>1 Gy) is an established human carcinogen, with a linear relationship to cancer risk (1, 2). Its major mechanism of carcinogenicity is DNA damage (1, 2). In contrast, risk estimates from epidemiological studies examining low radiation doses (<0.2 Gy) suffer from a significant uncertainty (3, 4). The biological effects of low-dose radiation differ from those observed at high doses and are mostly unrelated to DNA damage *per se* (5–13). In the absence of reliable estimates, the conventional approach extrapolates the linear trend observed at high-dose radiation to low-dose exposures, which is known as the “linear no-threshold hypothesis” (LNTH) (1, 14–17). Such extrapolation has been criticized, because it assumes the same carcinogenic mechanism as operating at high doses, whereas the biological phenomena are specific to low-dose exposure. These effects can be summarized as three competing hypotheses. The bystander effect hypothesis predicts a risk greater than expected according to the LNTH, because irradiated cells can transmit radiation effects to un-irradiated neighboring cells (9–13, 16, 18, 19). The threshold hypothesis considers radiation-induced cancer risk at low doses to be close to zero due to a threshold of radiation-induced damaging effects (17, 20, 21). Finally, the adaptive response hypothesis considers a beneficial effect of low-dose radiation exposure via induction of defense mechanisms that may improve resilience to hazardous exposures overall (21–24). We believe that all three phenomena might present a complex response to low-dose radiation and these hypotheses are not mutually exclusive. In that case, relative intensity of these responses may differ between individuals; and the predominance of one or a balance between several response types would classify an individual as being extremely sensitive, resistant, or even benefiting from low-dose radiation exposure. On a population level, the risk estimates then would depend on the proportions of highly sensitive and resistant individuals, which in fact, may result in a linear no-threshold relationship with cancer risk (or other health effects) observed statistically. However, biologically such linear relationship may represent a mix of different individual responses within human populations.

It has been argued that the adherence to LNTH is useful as it represents a conservative approach to the risk–benefit analysis (25). However, this might be the case if LNTH is compared to either the threshold or the adaptive response hypotheses. When one considers the bystander effect scenario, reliance in policy-making on the LNTH-based model does not provide adequate protection to individuals with high radiosensitivity. Indeed, enhanced radiosensitivity has been documented among children and individuals with familial cancer predisposition (26–29). Therefore, protection of individuals and groups with high radiosensitivity requires adoption of even more stringent guidelines.

On the other hand, the proponents of the threshold/adaptive responses argue that the existing (conservative) approach has negative economical and societal implications and, therefore, should be relaxed. It was said that LNTH model “imposes excessive costs on the society,” “inspires radiophobia” resulting in “refusal of some patients to undergo potentially life-saving medical imaging,” and in “discouragement of the studies of low-dose radiation therapies”; moreover, it “provides motivation for radiological terrorism” (30). From either point of view, the LNTH model for radiation risk–benefit analysis seems to be inadequate.

## Individualized Approach to Radiation Safety at Low-Dose Exposure Promises to Avoid Both Enhanced Risks Among the Radiosensitive Subgroups and An Excessive Economic Burden of an Overly Strict Regulations

Individualized approach to radiation safety has an immediate practical implication affecting a large part of the general population in the field of medical imaging. The widespread use of computerized tomography (CT) scans has greatly increased public exposure to ionizing radiation (31). It has been estimated that over 70 million/year CT procedures may cause as many as 30,000 new cancer cases in the US alone (32). Because of its superior diagnostic value (33), there is no doubt that the use of CT scans will continue to grow. In this situation, knowing individual radiosensitivity can help to make an informed decision on the risk–benefit ratio in treatment of those who are very sensitive to radiation (34). Similar logic can be applied to other medical and non-medical situations that involve ionizing radiation exposure. For example, evacuation of residents from areas of low-dose exposure due to radiologic accidents may cause “non-radiogenic disaster-related premature deaths,” such as “officially registered among the evacuated population” in Fukushima (30). Individual approach provides little help in identification of most sensitive individuals after a nuclear catastrophe; however, screening of the population working on and living around nuclear power facilities can inform the urgency of evacuation from the areas with low-dose ionizing radiation (LDIR) exposure. Another example of areas where individualized approach can guide the risk–benefit analysis is testing candidates for spacecraft missions or atomic submarine crews.

## Individual Variability in Response to Low-Dose Radiation has been Documented

The phenomenon of individual differences in radiosensitivity has been known since the beginning of the twentieth century (35) and extensively reviewed in previously published reports (28, 36–38). However, the majority of previous studies examined human radiosensitivity to relatively high radiation doses. Regarding LDIR, studies of individual responses are less numerous and inconclusive. Here, we discuss the main advantages and limitations of these studies with the intention to pinpoint specific barriers to establishing population-based research in this area.

It has been suggested that cell and cellular processes are the main targets of the LDIR effects. Naturally, an analysis of individual variability should involve a comparison of cellular responses to LDIR in different individuals. Such a comparison can be conducted using various experimental designs. For example, *in vivo* biodosimetry studies estimate the frequency of binucleated cells in peripheral blood lymphocytes, comparing individuals with different levels of exposure (39). Biodosimetry studies may help to detect the level of radiation exposure in human population. However, radiation dosimetry may not be appropriate to predict the risk associated with LDIR in un-irradiated population or help to decide whether a substitution of a radiation-based imaging technique should be prescribed to one or another individual. In a more direct manner, individual *in vivo* response to irradiation was studied by Goldberg et al. (40). Specifically, a small area of skin was irradiated and then a skin biopsy was analyzed for transcriptomic responses. This study yielded two important results: it established that measurable cellular changes occur in the intact human tissues in response to a single low-dose radiation exposure and confirmed the great individual variability of such response. In this setting, individual variability can arise both from genetic predisposition that determines intrinsic radiosensitivity and individual exposures to different factors. For example, differences in diet (41–43), tobacco use (44, 45), or prescribed medications (46) can affect individual response (47). Such confounding background factors cannot be easily controlled, compromising comparisons of radiation responses between individuals *in vivo*. Another disadvantage of the *in vivo* approach relates to the main goal of such studies, i.e., to predict potential health risks associated with low-dose radiation. The study by Goldberg et al. (40) was conducted among prostate cancer patients scheduled to undergo radiotherapy, i.e., the study population that was to be exposed to higher radiation doses. Obviously, such a direct *in vivo* radiation test cannot be applied to the general population and especially to individuals suspected to be very sensitive to ionizing radiation.

Alternatively, individual radiosensitivity can be detected using primary cell cultures isolated from different individuals. This approach most importantly evades ethical concerns related to direct irradiation of human subjects. Furthermore, such study design allows a better control of the background factors via standardization of cell culture conditions.

Traditionally, primary cultures of peripheral blood lymphocytes, fibroblasts, or keratinocytes were used as models to study individual response to low-dose radiation (36, 37, 48). These studies definitely established that even a single very low dose of radiation exposure (<0.1 Gy) can produced a cellular response. Measurements of the DNA-related effects focused on DNA-damage response (49, 50) and alterations in chromatin structure (51). Others studied gene expression (8, 52–56), and proteomics (57). Similarly to the studies *in vivo*, this line of research demonstrated a significant individual variability of cellular responses but has not identified a unified measure of such response that can be used to rank individuals in their radiosensitivity. The main problem with the DNA-targeted and transcriptional responses is their transient nature, which complicates their application to population studies. In addition, these traditional cellular models have limitations either due to low-proliferation potential (primary lymphocytes) or the invasive process of primary cell procurement (as for fibroblasts or keratinocytes).

We would like to note that donor-specific immortalized cell lines also address the concerns related to controlling the background conditions and the ethical concerns of *in vivo* irradiation. These are good models for studying various cellular functions, except the mechanisms related to cell proliferation control, because immortalization of cells impacts natural mechanisms controlling cell proliferation, and specifically, the signaling pathways responsible for the cell cycle checkpoints. Therefore, donor-specific immortalized cell lines are not suitable for studying potential pro-carcinogenic or anti-carcinogenic responses to low-dose radiation that are related to cell proliferation control.

To summarize, advances in this research require a new cellular model that satisfies two major requirements: (a) cells can be non-invasively obtained on a population-based scale and (b) produce long-term primary cultures. Such a cellular model would permit search for a non-transient measurable response to low-dose radiation that can rank individuals in their radiosensitivity.

## Search for a Unified Measurement to Rank Individuals in Sensitivity to the Effects of Low-Dose Radiation

The ultimate goal is to develop a test that can be applied to a non-invasively obtained biological sample to assist a medical or a policy decision in risk–benefit analysis, radiation protection, or other scenarios. Application of such test to clinical practice can be similar to the routinely used allergy or antibiotic sensitivity tests. Also, because radiation protection policy often relies on epidemiological data, a radiation sensitivity test would be an important epidemiological tool allowing risk stratification and thereby leading to more precise estimation of risks associated with exposure to low-dose radiation.

This review does not present a comprehensive examination of the existing assays that can be adapted to test individual sensitivity to LDIR. We rather illustrate the concept of cell-based radiation sensitivity testing by presenting several promising approaches. An example could be formation of dicentric chromosomes and gamma-H2AX foci in lymphocytes of human blood samples following exposure to a CT scanner as was shown by Golfier et al. (49). This study found that gamma-H2AX foci formation presents a more sensitive readout as compared to quantification of dicentric chromosomes. However, the transient character of the former would preclude the adoption of such an assay in a population-based testing; in contrast, the persistence of dicentric chromosome formation may present a more promising readout for the development of a test. The next step would be evaluating the ability of this assay to distinguish individual differences in response to low-dose radiation. Similarly, other measurements related to the cytokinesis-block micronucleus cytome assay in lymphocytes, such as micronuclei (scoring chromosome breakage and/or whole chromosome loss) and nuclear buds (scoring gene amplification) (58) could present the basis for the development of a test, if they prove to be sensitive enough to reveal inter-individual differences. However, it is important to note that these assays are related to DNA-damage effects, whereas LDIR effect on DNA is not substantial. Hence, the development of non-DNA-related cell-based assays is critically needed for testing individual response to LDIR.

In general, the non-DNA-targeted approaches can be classified into three main lines of research and ranked by their ability to produce interpretable information. The “omics” approach including comprehensive analysis of transcriptional and proteome profiles together with dynamic change in metabolome and epigenetics presents a good starting point in cataloging individual responses to LDIR (59). However, the main weakness of this approach is interpretation of the data across different analytical platforms and high variability of results related to assay execution and biospecimen handling conditions (60). As noted by the NCI scientists, so far this approach did not generate data that would clearly lead to the development of any test (60).

Collectively, the “omics” studies suggest that relatively long-lived (>24 h) changes in intracellular signaling should be evaluated in LDIR-related cellular responses. Studies focused on the analysis of certain signaling pathways confirmed that LDIR exposure indeed results in long-lived changes in intracellular signaling (60–63). However, a measurable effect that can serve as a biomarker of individual differences in response to low-dose radiation remains to be identified.

Finally, some integrated measurements of cellular functions can lead to a desired outcome. As an example, induction or redirection of cellular differentiation can be studied as an anti- or pro-carcinogenic response, respectively (64, 65). This phenomenon has been studied in hematopoietic stem and progenitor cells, which are present at low frequency (<0.5% of mononuclear cells) in peripheral and umbilical cord blood. These cells can be identified by the expression of a transmembrane protein CD34 (CD34+ cells) and their differentiation can be followed by appearance of lineage-specific surface cell markers, thus, allowing quantification of cell differentiation response using blood specimens (66). Monzen et al. used purified CD34+ from cord blood to study their differentiation in response to different types and doses of ionizing radiation (66). This study did not find a difference in the total number of mononuclear cells generated in the culture of purified CD34+ cells in response to 0.5-Gy X-ray exposure but the fraction of different lineages has changed: the fraction of cells with eosinophil/neutrophil lineage markers decreased, whereas the erythroid-related lineage fraction increased. This example demonstrates that the analysis of cell differentiation response may present the basis for the development of individual radiosensitivity test.

In summary, cell-based research demonstrated that cellular response to LDIR *in vitro* can be detected. *In vitro* donor-specific cellular models have a potential to evolve into an individual radiosensitivity test. However, there is an important caveat: these models and assays should reveal inter-individual variability, be sensitive to detect the response at a low dose, and be amenable to high-throughput screening instrumentation and protocols (67).

## At the Intersection of Epidemiology and Cell Biology: Our Experience with a Model for Individual Response to LDIR

An epidemiological cell-based model should (a) be donor-specific, (b) allow for robust *in vitro* culturing, thus, amenable for high-throughput screening, (c) produce highly viable cultures after cryopreservation, and (d) be obtained in a least invasive manner.

Blood-derived CD31+/CD34+ endothelial colony-forming cells (ECFCs) were found to fulfill these requirements. ECFCs form highly proliferative cell cultures originating from a single cell, which forms a colony of progenitors (hence, the name) under certain growth conditions. These cells can be propagated over many passages maintaining endothelial phenotype (68). We found that ECFCs can be cryopreserved for many months and even years. Therefore, we isolated ECFCs from cord blood of three donors to search for quantifiable cellular responses to low-dose radiation (69). We found that a single radiation dose of 0.05 Gy significantly inhibits cell proliferation, a response that is not observed in immortalized cell lines at such a low dose. The response appears to be donor-specific (69). Importantly, this LDIR effect appeared only 48 h post-irradiation. We have recently extended our observation to even lower dose of 0.01 Gy (data not published). We did not find any indication of ECFCs’ death after irradiation. Thus, LDIR inhibits ECFCs proliferation but does not kill the cells suggesting that such a treatment promotes either cell senescence or differentiation.

Our experience working with donor-specific ECFCs as a model lead to several important conclusions. First, these cells are very sensitive to low-dose radiation. Second, non-DNA-based readouts such as proliferation can be used to analyze response to LDIR because they can persist for several days. Third, the effects of LDIR can be quantified in cell cultures that are donor-specific, thus, providing the bases for individual testing. Although this research has been only recently initialized and is still at its infancy, it indicates that donor-specific radiation sensitivity testing is achievable.

## Conclusion

The LNT model of the health risk associated with low-dose radiation has been criticized on the basis of different biological effects induced by high- vs. low-doses of radiation. However, whether the departure from linearity is toward increased or decreased risk presents a hot point of the debate in the field of radiation risk–benefit analysis and radiation protection. We hypothesize that both increased and decreased risks are possible depending on the individual response to LDIR. In fact, there is a consensus that individuals differ in their radiosensitivity. Although it has been recognized that the main effects of LDIR are not related to DNA damage *per se*, the initial search for indicators of individual radiosensitivity has been rooted in quantification of DNA-damage responses. The low levels and the transitory nature of DNA-damage related indicators demonstrated the futility of this approach. The next phase in this research addressed the question of which genes, proteins, and pathways are responsible for specific responses to LDIR. This phase entailed screening different “omics” platforms to catalog biological responses induced by LDIR and confirmed first, that many effects are transitory and second, that individual responses vary indeed. However, these approaches could not capture quantifiable indicators of individual responses. Currently, this search is focused on cellular models that can be donor-specific and satisfy the non-invasive requirement of cell procurement. We share our experience working with blood-derived donor-specific ECFCs as a model to find an indicator of individual radiosensitivity. Other assays using blood-derived cells confirm that this can be a promising approach. The goal now is to find a quantifiable measure of cellular response amiable to high-throughput population testing that is sensitive enough to rank individuals by their radiosensitivity.

## Conflict of Interest Statement

Alexander Kinev is a founder and executive of Creative Scientist, Inc. He is also a majority shareholder of this company, which develops a radiation sensitivity test. No other conflicts of interest are reported. This research was supported by an epidemiology grant from the National Brain Tumor Society.
